# De novo variant analysis of childhood-onset obsessive-compulsive disorder in the French-Canadian population

**DOI:** 10.1038/s41398-025-03661-4

**Published:** 2025-10-31

**Authors:** Kate Bornais, Jay P. Ross, Zoe Schmilovich, Miranda Medeiros, Charles-Étienne Castonguay, Dan Spiegelman, Bernard Boileau, Jean-Jacques Marier, Ghislain Laurin, Patrick A. Dion, Guy A. Rouleau

**Affiliations:** 1 Department of Human Genetics, McGill University, Montréal, QC Canada; 2 Montréal Neurological Institute-Hospital, McGill University, Montréal, QC Canada; 3 Department of Neurology and Neurosurgery, McGill University, Montréal, QC Canada; 4 Le Centre Hospitalier Universitaire Sainte-Justine, Université de Montréal, Montréal, QC Canada

**Keywords:** Genetics, Psychiatric disorders

## Abstract

Childhood-onset obsessive-compulsive disorder (OCD) is a neuropsychiatric disorder with a strong genetic component. De novo variants (DNVs) have been shown to have a role in childhood-onset OCD, but to date, no DNV analysis has been performed in patients from a genetically isolated population. Here, we aimed to investigate the impact of rare de novo single nucleotide variants (dnSNVs) on childhood-onset OCD risk in the French-Canadian population. In a cohort of 36 French-Canadian trios comprised of 36 probands with childhood-onset OCD and 72 unaffected parents, we identified 34 dnSNVs harboured in 34 different genes. We found that four of these genes were previously associated with OCD, replicating their contribution to its risk. We also observed complete overlap between our 34 candidate genes and genes associated with 11 related neuropsychiatric disorders, supporting a shared underlying genetic susceptibility across psychopathologies. Among genes harbouring DNVs across three childhood-onset OCD cohorts, we observed an overrepresentation of genes involved in clathrin-dependent endocytosis (GO:0072583; *p-adj* = 0.0498) and phosphatidylinositol binding (GO:0035091; *p-adj* = 0.0431), offering potential biological mechanisms underlying childhood-onset OCD. No association was found between the number of dnSNVs in childhood-onset OCD probands and OCD symptom severity. Altogether, this study offers a framework for performing DNV analyses of complex disorders in genetically isolated populations. Additionally, we have provided the first list of candidate childhood-onset OCD genes in the French-Canadian population.

## Introduction

Obsessive-compulsive disorder (OCD) is a common neuropsychiatric disorder with a lifetime prevalence of ~1–3% in the general population [1]. Individuals with OCD experience recurrent intrusive thoughts or sensations (obsessions) and, in response, excessively perform repetitive behaviours (compulsions) aiming to alleviate their anxiety [2]. Symptoms of OCD can cause such substantial distress and/or impairment in crucial areas of functioning that the World Health Organization currently ranks OCD among the top 10 most debilitating disorders of any kind [3, 4].

Childhood-onset OCD represents a clinically distinct patient subgroup of OCD characterized by the manifestation of symptoms before 18 years of age [5]. While the aetiologies of both childhood- and adult-onset OCD are multifaceted and remain poorly understood, studies focused on one subtype allow for a more targeted investigation of the genetic landscape of OCD and can uncover genetic factors contributing to the distinct clinical trajectory of a particular subgroup. Childhood-onset OCD, as compared to adult-onset OCD, is associated with greater global symptom severity and is predicted to have a stronger genetic component, with heritability estimates being ~45–65% for childhood-onset OCD and ~27–45% for adult-onset OCD [1, 5, 6]. Therefore, childhood-onset OCD cohorts are better suited for genetic investigations.

De novo variants (DNVs) are rare genomic alterations that arise in parental germ cells (eggs or sperm) or zygotes (fertilized eggs) but are not present in the genomes of either biological parent [7, 8]. DNVs are frequently more deleterious than inherited variants due to their lack of evolutionary selection and typically impart large phenotypic effects [8, 9]. As such, DNV approaches have shown great success for systematic risk gene discovery in complex neuropsychiatric phenotypes [9, 10]. Given that DNVs are predicted to play a larger role in severe early-onset psychiatric disorders than late-onset disorders, DNV analyses are especially valuable for investigating the genetic basis of childhood-onset psychopathologies [7].

The role of rare DNVs in risk for childhood-onset OCD is well-established [11, 12]. It has been estimated that DNVs contribute to risk in ~22% of childhood-onset OCD cases [11, 12]. A significant exome-wide enrichment of damaging DNVs has also been previously observed in cases with childhood-onset OCD relative to their unaffected parents and general population controls [11, 12]. Additionally, two high-confidence risk genes—chromodomain-helicase-DNA-binding protein 8 (*CHD8*) and signal peptide, CUB domain and EGF like domain containing 1 (*SCUBE1*)—were identified in childhood-onset OCD through DNV analyses of whole-exome sequencing (WES) data of parent-child trios [11, 12]. However, the number of DNVs in childhood-onset OCD remains limited and many of the associated genes have yet to be replicated.

To date, no DNV study of childhood-onset OCD has been performed in a genetically isolated founder population. These populations are characterized by a limited gene pool and a high degree of shared ancestry. Studying DNVs within genetically isolated populations offers an opportunity to uncover novel genetic contributors to disorders that may have arisen independently within specific groups [13]. Additionally, using genetically homogenous populations for DNV analyses can increase confidence in calling true DNVs. This is due to the phenomenon of “missed heterozygotes”, wherein heterozygous variants can be wrongly sequenced as absent from a parent due to technical or computational errors, leading to apparent DNVs in children that were actually inherited [14, 15].

The French-Canadian population of Québec, Canada, is a widely recognized genetic isolate that has yielded many findings that other large-scale studies would have missed due to population background heterogeneity [16]. The historical founder effect of the French-Canadian population and their subsequent isolation due to linguistic, religious, and geographical barriers in the 17^th^ century resulted in a distinct and homogenous genetic background that requires its own complete catalogue of disorder-associated variants [16]. The French-Canadian population also often allows access to familial data, typically with data available from both parents, and is deeply clinically phenotyped. Considering OCD is one of the most prevalent psychiatric disorders in Québec, with a provincial prevalence of ~1.2% [17], a DNV analysis of French-Canadian cases with childhood-onset OCD represents an important avenue of investigation.

Here, we performed a DNV analysis of childhood-onset OCD probands and their unaffected biological parents from the French-Canadian population to identify genetic factors associated with childhood-onset OCD risk and symptom severity within the population. In doing so, we have developed a framework for conducting DNV analyses of complex psychiatric disorders in genetically isolated populations.

## Materials and methods

**Fig. 1:**
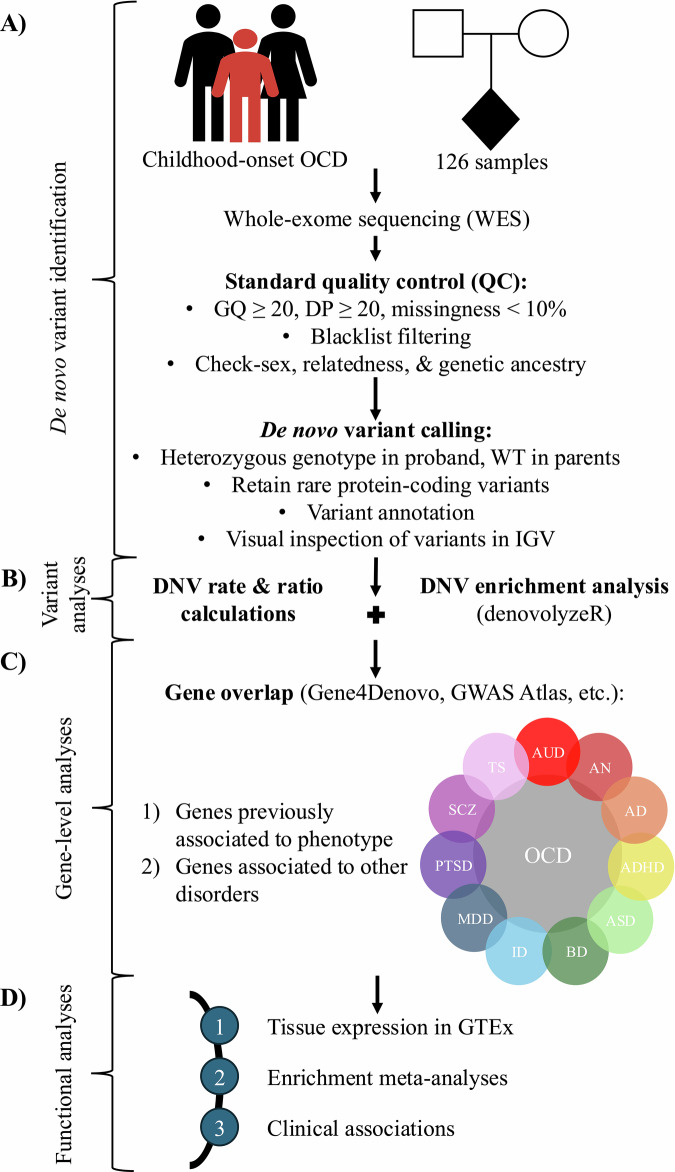
A framework for performing DNV analyses of complex psychiatric disorders in genetically isolated populations. **A** The discovery flow of DNVs from trios, including sample collection, standard data QC, DNV calling and annotation, and visual inspection of DNVs. **B** Variant-level analyses, including de novo mutation rate and ratio calculations as well as enrichment analyses of variant classes. **C** Gene-level analyses, including examining for overlap within and between phenotypes using publicly available databases. **D** Functional analyses, including tissue expression and enrichment analyses of candidate genes, with the enrichment meta-analyses incorporating data from previous studies (e.g., Cappi et al., 2020 [11]; Halvorsen et al., 2021 [12]), as well as correlational analyses of DNVs with clinical data.

The methodological framework presented below can be visualized in Fig. 1.

### Cohort overview

In total, 42 complete trios consisting of 42 probands with childhood-onset OCD and 84 unaffected parents from Québec, Canada, were collected as part of this study (*n* = 126). Initial inclusion criteria required all three trio participants to have self-reported as French Canadian. We excluded six trios where one or more members self-reported as another ancestry. Samples underwent 100x WES at Axeq Technologies MacroGen Laboratory (Rockville, Maryland) with the SureSelect XT Human All Exon enrichment kit version 7 (Agilent) using the Illumina NovaSeq6000 S4 Sequencing System and following standard protocols. Reads were aligned to the *Homo sapiens* (human) reference genome build GRCh37 (hg19). All OCD diagnoses were made by psychiatrists at the Centre Hospitalier Universitaire Sainte-Justine (Montréal, Québec, Canada) and were based on the Diagnostic and Statistical Manual of Mental Disorders, fourth edition (DSM 4) criteria [18]. Additional inclusion criteria required probands to have received their OCD diagnosis before 18 years of age and have no known history of OCD in first-degree relatives. Cohort and sample details are summarized in Supplementary Tables 1 and 2, respectively.

### Quality control

Variant- and sample-level quality control (QC) were performed using the Genome Analysis Toolkit (GATK) version 3.8 (Supplementary Table 3) [19]. Variants were filtered according to the following standard QC thresholds: genotype quality (GQ) ≥ 20, depth of coverage (DP) ≥ 20, and missingness < 10% [11, 12]. Variants in blacklist regions of the genome were also removed [20]. Biological sex was imputed for each sample to ensure proper sample identity using the PLINK2.0 *--check-sex* function [21]. A kinship analysis was then conducted using the KING algorithm to ensure that trios displayed biological parent-child relationships, parents were in non-consanguineous relationships, and families were unrelated [22]. Principal component analysis (PCA) was performed to cluster individuals by genetic ancestry and account for population stratification [23]. WES data of each participant were compared to the 1000 Genomes reference dataset to filter out samples that did not cluster with the expected European genetic ancestry and to ensure tight clustering indicative of a genetically homogenous study population [24].

### De novo variant calling, annotation, and filtration

The initial set of DNVs was filtered for heterozygous genotype in the proband and wildtype in both parents using the GATK version 3.8 [19], and then annotated using the Ensembl Variant Effect Predictor (VEP) [25]. DNV calls were included in the downstream analyses if they were protein-coding, observed only once in the cohort, and had a minor allele frequency (MAF) < 1% in the non-Finnish European (NFE) reference population of the Genome Aggregation Database (gnomAD) version 4.1.0 [9, 11, 12, 26]. Missense, nonsense, splice site, and synonymous single nucleotide variants (SNVs) were retained. All de novo SNV (dnSNV) calls passing these criteria were visually inspected in the Integrative Genomics Viewer (IGV). Any call detected in < 30% of proband reads or >5% of parent reads at the call site were removed [12]. Variants that displayed strong strand biases and/or false positives due to misalignment of reads or low base quality (Phred Quality Score (Q) < 20) were also removed [27]. To ensure individual sample quality, exclusion of probands with more than 5 dnSNV calls meeting these criteria was considered, but no probands were excluded at this QC step as none exceeded the threshold [11, 12]. The deleteriousness of the final set of dnSNVs was determined based on their Combined Annotation Dependent Depletion (CADD) score [28].

### De novo mutation rate and probability analyses

We calculated the average number of dnSNVs per proband, from which we estimated the observed de novo mutation rate of the French-Canadian childhood-onset OCD probands. The observed de novo mutation rate was calculated as follows:

$${\text{De}}\,{\text{novo}}\,\mathrm{mutation\; rate}=(\mathrm{number\; of\; dnSNVs}/\mathrm{number\; of\; probands})\,/(\mathrm{size}\,\mathrm{of}\,\mathrm{RefSeq}\,\mathrm{hg}19\,\mathrm{coding\; exome\; x\; number\; of\; gametogeneses})$$

where the size of the *Homo sapiens* (human) reference genome (RefSeq) GRCh37 (hg19) coding exome was 33,828,798 base pairs (bps) [29] and the number of gametogeneses was two (spermatogenesis and oogenesis). We also calculated the average number of non-synonymous and synonymous dnSNVs per proband to determine the observed ratio of non-synonymous to synonymous dnSNVs. Non-synonymous and synonymous dnSNV counts were then compared using a Poisson exact test (overdispersion test: *p* > 0.05) with the *poisson.test* function from the base R package.

To assess whether there was a genome-wide excess of dnSNVs in different functional variant classes, we used the *denovolyzeByClass* function from the denovolyzeR R package [30]. Briefly, denovolyzeR implements a mutational model that estimates the probability of a dnSNV occurring in a single copy of each human gene in one generation based on the local sequencing context, from which it can calculate the expected number of dnSNVs for a given population size [30]. The *denovolyzeByClass* function tabulates the dnSNV probabilities for each variant class (e.g., nonsense, missense, splice site, synonymous, etc.). It then determines whether there is an overrepresentation of dnSNV counts in a particular variant class within a population of interest by comparing the expected dnSNV frequencies to those observed using a Poisson framework [30]. For each variant class, the function returns the expected number of dnSNVs, the enrichment ratio (= observed/expected), and *p*-values obtained from the Poisson regression [30].

### Gene-level analyses

Genes harbouring dnSNVs that passed all QC criteria were gathered to generate a list of candidate childhood-onset OCD genes. The literature was mined to identify genes that have been previously associated with OCD through other DNV analyses or genome-wide association studies (GWASs) [11, 12, 31]. We then compared these genes with our list of candidate childhood-onset OCD genes to identify overlap. Additionally, due to the known clinical and genetic overlap across psychiatric disorders, we assessed whether our candidate childhood-onset OCD genes were also associated with related psychiatric disorders. We compared our candidate gene list to genes previously associated with 11 other psychiatric disorders through DNV analyses listed in the Gene4Denovo database and GWASs listed in the GWAS Atlas, respectively [32, 33]. To confirm that our candidate genes were expressed in the brain, we input our candidate gene list into the Expression feature of the Genotype-Tissue Expression (GTEx) Project Portal [34]. Using the Multi Gene Query of this feature, we performed a multi-gene search for expression across all available brain tissues [34]. This output a heatmap representing normalized transcript per million (TPM) expression values from RNA-seq data for each gene in the selected tissues [34].

### Functional enrichment meta-analyses

Genes harbouring dnSNVs in our French-Canadian childhood-onset OCD probands (*n* = 34 genes) were combined with genes harbouring DNVs in 771 previously reported OCD trios and 41 previously reported OCD quartets (*n* = 871 genes) [11, 12]. The combined list of candidate childhood-onset OCD genes (*n* = 905 genes) was input into EnrichR and g:Profiler g:GOSt for functional enrichment meta-analyses [35, 36]. Three enrichment analyses were conducted: 1) enrichment of Gene Ontology (GO) biological process terms; 2) enrichment of GO molecular function terms; and 3) enrichment of All RNA-seq and ChIP-seq Sample and Signature Search (ARCHS^4^) tissue terms [37, 38]. To account for multiple comparisons within each analysis, *p*-values for term enrichment were adjusted using Benjamini–Hochberg false discovery rate (BH-FDR) correction. BH-FDR-adjusted *p*-values (*p-adj*) < 0.05 were considered statistically significant. Results from the two sources were compared and only common pathways identified by both EnrichR and g:Profiler g:GOSt to be significantly enriched after BH-FDR correction were considered.

### Statistical analyses

To control for multiple comparisons within each model below, *p*-values for all input variables were adjusted using the BH-FDR method available in the *p.adjust* function from the base R package. BH-FDR-adjusted *p*-values (*p-adj*) < 0.05 were considered statistically significant.

Parental ages at proband conception were calculated by subtracting proband birthdates from parent birthdates for 32 of the 36 fathers and 31 of the 36 mothers retained following QC. We then used two linear regression models to assess the relationship between the number of dnSNVs in our French-Canadian probands and parental ages at proband conception, while adjusting for proband biological sex and the first five ancestry principal components (PCs) as covariates:

$${\rm{dnSNV\; number}} \sim {\rm{father\; age\; at\; conception}}+{\rm{covariates}}$$

$${\rm{dnSNV\; number}} \sim {\rm{mother\; age\; at\; conception}}+{\rm{covariates}}$$

Age at OCD diagnosis was available for all 36 probands retained following QC. We used a linear regression model to assess the relationship between the number of dnSNVs in probands and proband age at OCD diagnosis, while adjusting for proband biological sex and the first five ancestry PCs as covariates:

$${\rm{age\; at\; OCD\; diagnosis}} \sim {\rm{dnSNV\; number}}+{\rm{covariates}}$$

To ensure linear regression assumptions were met, residuals were tested for normality (Shapiro-Wilk test: *p* > 0.05) and inspected via Q-Q plots. Homoscedasticity was confirmed through visual inspection of residuals versus fitted values, and multicollinearity was assessed (Variance Inflation Factors (VIFs) < 5).

Finally, we used a logistic regression model to examine the effect of the number of dnSNVs in our French-Canadian probands on whether OCD symptoms were rated as severe or mild to moderate, while adjusting for age at OCD diagnosis, biological sex, and the first five ancestry PCs as covariates:

$${\rm{symptom\; severity}} \sim {\rm{dnSNV\; number}}+{\rm{relatives}}+{\rm{brain\; trauma}}+{\rm{comorbidity}}+{\rm{covariates}}$$

The severity of obsessive and compulsive symptoms was measured using the Children’s Yale-Brown Obsessive Compulsive Scale (CY-BOCS) for 30 out of the 36 included probands at the time of their diagnoses [39]. Total CY-BOCS scores were calculated as the sum of the obsessions and compulsions subscale severity scores [39]. Probands with CY-BOCS scores ≥ 24 were classified as having severe OCD symptoms, while probands with CY-BOCS scores < 24 were classified as having mild to moderate OCD symptoms [39].

Whether probands have first-degree relatives with a psychiatric diagnosis was included in the model to control for inherited genetic risk factors; history of traumatic brain injury was included in the model to control for a known environmental risk factor in childhood-onset OCD; and presence of a comorbid neuropsychiatric disorder was included in the model to control for the general liability to psychopathologies. To ensure logistic regression assumptions were met, multicollinearity was evaluated (VIFs < 10), and good model fit was assessed with McFadden’s R² (>0.4).

We conducted post hoc power analyses using the pwr R package to determine the minimal detectable effect size (Cohen’s f^2^) for all regression models, assuming a significance level (α) of 0.05, a desired power (β) of 0.8, and a sample size (N) of 32, 31, 36, and 30 probands for the respective models.

## Results

Following QC, 36 trios (36 probands and 72 unaffected parents) were included in the analyses. Trio probands were 53% female with a mean age at diagnosis of 13.0 ± 2.48 years.

### dnSNVs detected in French-Canadian childhood-onset OCD probands

Our framework directly identified 34 dnSNVs in 34 genes across the 36 French-Canadian childhood-onset OCD probands (0.94 dnSNVs/proband, average CADD score = 16.1) that remained after QC (Table 1; Supplementary Table 4). Ten probands (27.8%) harboured one dnSNV, nine (25.0%) harboured two dnSNVs, two (5.6%) harboured three dnSNVs, and 15 (41.7%) had no dnSNVs detected (Table 1; Supplementary Table 4). 17 of the 34 (50.0%) dnSNVs were missense variants, three (8.8%) were splice site variants, and 14 (41.2%) were synonymous variants (Table 1; Supplementary Table 4). 13 of the 34 (38.2%) dnSNVs were variants ranking in the top 1% of deleterious variants in the human genome (i.e., had a CADD score > 20) (Table 1; Supplementary Fig. 1) [28]. The estimated de novo mutation rate in our French-Canadian childhood-onset OCD probands was 1.40 × 10^−8^ per nucleotide per generation (95% CI: 9.36 × 10^−9^, 1.86 × 10^−8^). The observed ratio of non-synonymous to synonymous dnSNVs was 1.43:1 (0.56 non-synonymous dnSNVs/proband, 0.39 synonymous dnSNVs/proband). We observed no difference between the counts of non-synonymous dnSNVs and synonymous dnSNVs in our 36 childhood-onset OCD probands (*p* = 0.108, 95% CI: 0.873, 2.21). All 34 of the candidate childhood-onset OCD genes were predicted to be expressed in one or more brain tissues (Supplementary Fig. 2) [34].

**Table 1:** De novo single nucleotide variants in French-Canadian childhood-onset OCD probands.

Proband	Position (hg19)	Position (hg38)	HGVS Nomenclature	Variant Class	Gene Symbol	gnomAD NFE AF v4.1.0	CADD PHRED Score
TOC065	chr5:5237111 G > A	chr5:5236998 G > A	NM_139056.4:c.2053 G > A(p.Ala685Thr)	Missense	*ADAMTS16*	3.98E-05	29.8
TOC056	chr15:85400980 C > T	chr15:84857749 C > T	NM_020778.5:c.3011 C > T(p.Ala1004Val)	Missense	*ALPK3*	Absent	26.4
TOC007	chr2:202154268 C > T	chr2:201289545 C > T	NM_001127391.3:c.1054 G > A(p.Glu352Lys)	Missense	*ALS2CR12*	Absent	25.1
TOC070	chr17:27934828 T > C	chr17:29607810 T > C	NM_152345.5:c.183 T > C(p.Leu61 = )	Synonymous	*ANKRD13B*	Absent	10.54
TOC026	chr11:118772435 G > A	chr11:118901726 G > A	NM_001378213.1:c.2017C>T(p.Arg673Trp)	Missense	*BCL9L*	5.93E-06	19.18
TOC018	chr9:136898768 C > T	chr9:134033646 C > T	NM_007371.4:c.2125 G > A(p.Glu709Lys)	Missense	*BRD3*	0.00	22.9
TOC049	chr2:29294791 C > T	chr2:29071925 C > T	NM_001029883.3:c.2337 G > A(p.Pro779 = )	Synonymous	*C2orf71*	1.27E-05	4.379
TOC047	chr9:132374730 A > T	chr9:129612451 A > T	NM_199350.4:c.1192 T > A(p.Ser398Thr)	Missense	*C9orf50*	8.48E-06	18.46
TOC034	chr19:3613091 C > T	chr19:3613093 C > T	NM_001080543.2:c.1751 G > A(p.Arg584His)	Missense	*CACTIN*	8.53E-07	31
TOC034	chr10:95275285 C > A	chr10:93515528 C > A	NM_018131.5:c.652 C > A(p.Gln218Lys)	Missense	*CEP55*	Absent	24
TOC015	chr6:55989036 C > T	chr6:56124238 C > T	NM_030820.4:c.1704+1 G > A	Splice site	*COL21A1*	8.53E-07	33
TOC069	chr7:91763562 G > A	chr7:92134248 G > A	NM_000786.4:c.117 C > T(p.Ala39 = )	Synonymous	*CYP51A1*	1.70E-06	14.27
TOC003	chr18:28649000 C > T	chr18:31069034 C > T	NM_024422.6:c.2368 G > A(p.Gly790Arg)	Missense	*DSC2*	0.00	22.6
TOC065	chr1:16641689 G > A	chr1:16315194 G > A	NM_018994.3:c.225 C > T(p.Cys75 = )	Synonymous	*FBXO42*	Absent	12.61
TOC017	chr11:49227684 A > G	chr11:49206132 A > G	NM_004476.3:c.159 T > C(p.Thr53 = )	Synonymous	*FOLH1*	8.48E-07	3.011
TOC002	chr7:1538340 C > T	chr7:1498704 C > T	NM_001080453.3:c.1283+3 G > A	Splice site	*INTS1*	Absent	1.151
TOC056	chr1:15421388 C > T	chr1:15094892 C > T	NM_201628.3:c.1506 C > T(p.Arg502 = )	Synonymous	*KAZN*	Absent	10.64
TOC032	chr2:23862064 G > A	chr2:23639194 G > A	NM_052920.2:c.341 G > A(p.Arg114Gln)	Missense	*KLHL29*	1.56E-04	27.8
TOC020	chr15:40678575 G > A	chr15:40386374 G > A	NM_033286.4:c.317 G > A(p.Arg106Gln)	Missense	*KNSTRN*	2.29E-05	24.2
TOC004	chr1:229636544 G > T	chr1:229500797 G > T	NM_018230.3:c.472 C > A(p.Leu158Ile)	Missense	*NUP133*	Absent	7.759
TOC039	chr1:228433346 C > T	chr1:228245645 C > T	NM_001386125.1:c.3990 C > T(p.Cys1330 = )	Synonymous	*OBSCN*	2.52E-03	0.761
TOC039	chr6:143795919 A > G	chr6:143474782 A > G	NM_003630.3:c.748-4 A > G	Splice site	*PEX3*	1.85E-05	8.787
TOC047	chr2:219205501 C > T	chr2:218340778 C > T	NM_015488.5:c.516 C > T(p.His172 = )	Synonymous	*PNKD*	0.00	12.52
TOC046	chr8:30890266 G > A	chr8:31032750 G > A	NM_001323311.2:c.33 C > T(p.Gly11 = )	Synonymous	*PURG*	7.33E-06	13.33
TOC046	chr20:20552120 T > C	chr20:20571476 T > C	NM_020343.4:c.3138 A > G(p.Gly1046 = )	Synonymous	*RALGAPA2*	Absent	11.94
TOC069	chr14:23817468 C > T	chr14:23348259 C > T	NM_016609.7:c.1073 G > A(p.Arg358Gln)	Missense	*SLC22A17*	2.46E-05	23.4
TOC003	chr1:48877209 G > A	chr1:48411537 G > A	NM_019073.4:c.332 C > T(p.Pro111Leu)	Missense	*SPATA6*	6.03E-05	27.9
TOC026	chr20:42089626 A > G	chr20:43460986 A > G	NM_006275.6:c.958 A > G(p.Lys320Glu)	Missense	*SRSF6*	Absent	22.6
TOC012	chr5:134785360 C > T	chr5:135449670 C > T	NM_001099221.2:c.270 G > A(p.Thr90 = )	Synonymous	*TIFAB*	3.64E-05	0.253
TOC005	chr8:23082421 G > T	chr8:23224908 G > T	NM_003844.4:c.154 C > A(p.Arg52 = )	Synonymous	*TNFRSF10A*	0.00	7.862
TOC007	chr8:100829947 G > C	chr8:99817719 G > C	NM_152564.5:c.8277 G > C(p.Leu2759 = )	Synonymous	*VPS13B*	Absent	7.632
TOC039	chr6:29640964 G > A	chr6:29673187 G > A	NM_001109809.5:c.924 C > T(p.Ile308 = )	Synonymous	*ZFP57*	7.62E-06	0.348
TOC070	chr16:88496000 G > A	chr16:88429592 G > A	NM_001367624.2:c.2122 G > A(p.Ala708Thr)	Missense	*ZNF469*	0.00	10.63
TOC003	chr1:247264596 C > T	chr1:247101294 C > T	NM_001142572.2:c.217 G > A(p.Glu73Lys)	Missense	*ZNF669*	0.00	15.16

34 dnSNVs were detected across 36 French-Canadian childhood-onset OCD probands. Ten probands (27.8%) had one dnSNV, nine (25.0%) had two dnSNVs, two (5.6%) had three dnSNVs, and 15 (41.7%) had no dnSNVs detected. 17 of the 34 (50.0%) dnSNVs were missense variants, three (8.8%) were splice site variants, and 14 (41.2%) were synonymous variants. 13 of the 34 (38.2%) dnSNVs had CADD scores > 20. These 34 dnSNVs were found to be harboured in 34 different genes. All annotations were made using the Ensembl VEP.

### A nominal genome-wide enrichment of splice site dnSNVs in French-Canadian childhood-onset OCD probands

Using denovolyzeR, we observed no enrichment (i.e., no more observed dnSNVs than expected) of missense dnSNVs (*p* = 0.908, BH-FDR = 0.908, enrichment ratio = 0.75) or synonymous dnSNVs (*p* = 0.143, BH-FDR = 0.215, enrichment ratio = 1.39) in our probands. However, we found a nominally significant enrichment (i.e., more observed dnSNVs than expected) of splice site dnSNVs (*p* = 0.0173, BH-FDR = 0.0519, enrichment ratio = 5.60).

### Four genes harbouring dnSNVs in French-Canadian childhood-onset OCD probands overlapped with genes previously associated to OCD

To increase confidence in the role of our candidate genes in childhood-onset OCD, we sought to identify repeated observations of DNVs in genes identified to harbour dnSNVs in our French-Canadian probands. Of our 34 candidate genes, two (5.9%)—integrator complex subunit 1 (*INTS1*) and TRAF-interacting protein with FHA domain-containing protein B (*TIFAB*)—were identified in another DNV analysis of WES data of childhood-onset OCD trios [12]. Additionally, two (5.9%)—spermatogenesis associated 6 (*SPATA6*) and zinc finger protein 669 (*ZNF669*)—overlapped with loci from the 2018 OCD GWAS (Table 2) [31].

**Table 2:** Genes harbouring dnSNVs in French-Canadian childhood-onset OCD probands with previously demonstrated associations to OCD and 11 related psychiatric disorders.

Psychiatric disorder	Genes previously associated with disorder	Genetic search space
OCD	*INTS1*^&^; SPATA6^*^; *TIFAB*^&^; ZNF669^*^	Common genomic variants^*^; rare protein-coding variants^&^
Alcohol-use disorder	*ADAMTS16*; *ALS2CR12*; *BRD3*; *CEP55*; *COL21A1*; *KLHL29*; *NUP133*; *PURG*; *RALGAPA2*; *SPATA6*; *TIFAB*; *TNFRSF10A*; *VPS13B*	Common genomic variants
Anorexia nervosa	*DSC2*	Common genomic variants
Anxiety disorder	*ALS2CR12; ANKRD13B*; *FOLH1*; *KNSTRN*; *VPS13B*	Common genomic variants
Attention deficit/hyperactivity disorder	*BRD3*; *C2orf71*; *CEP55*; *FOLH1*; *INTS1*; *KLHL29*; *PNKD*; *PURG*; *RALGAPA2*	Common genomic variants
Autism spectrum disorder	ALPK3^*^; ALS2CR12^*^; *BCL9L*^&^; *CACTIN*^&^; *COL21A1*^&^; *INTS1*^&^; *KAZN*^&^; KLHL29^*&^; KNSTRN^*^; *OBSCN*^&^; PURG^*^; *RALGAPA2*^&^; ZFP57^*^	Common genomic variants^*^; rare protein-coding variants^&^
Bipolar disorder	*ALPK3*; *ANKRD13B*; *BCL9L*; *BRD3*; *CEP55*; *COL21A1*; *DSC2*; *FOLH1*; *KAZN*; *PURG*; *RALGAPA2*; *SLC22A17*; *VPS13B*	Common genomic variants
Intellectual disability	*BRD3*	Rare protein-coding variants
Major depressive disorder	*ALPK3*; *ALS2CR12*; *BCL9L*; *BRD3*; *C9orf50*; *CEP55*; *CYP51A1*; *DSC2*; *FOLH1*; *OBSCN*; *PEX3*; *PNKD*; *TIFAB*; *VPS13B*; *ZNF469*; *ZNF669*	Common genomic variants
Post-traumatic stress disorder	*CACTIN*; *NUP133*	Common genomic variants
Schizophrenia	ALPK3^*^; BCL9L^*^; BRD3^*&^; C2orf71^*^; CACTIN^*^; CEP55^*^; COL21A1^*^; CYP51A1^*^; FBXO42^*^; FOLH1^*^; INTS1^*^; KAZN^*^; KLHL29^*^; KNSTRN^*^; *RALGAPA2*^&^; SLC22A17^*^; SRSF6^*^; TNFRSF10A^*^; ZFP57^*^	Common genomic variants^*^; rare protein-coding variants^&^
Tourette’s syndrome	*OBSCN*	Rare protein-coding variants

Two of the 34 (5.9%) candidate genes were previously associated with childhood-onset OCD by another DNV analysis of parent-child WES data. Additionally, two (5.9%) overlapped with loci identified in the 2018 OCD GWAS. Eight (23.5%) genes have been implicated in autism spectrum disorder, one (2.9%) in intellectual disability, two (5.9%) in schizophrenia, and one (2.9%) in Tourette’s syndrome by DNV analyses listed in the Gene4Denovo database. All 34 genes overlapped with loci identified in GWASs of nine related psychiatric disorders in the GWAS Atlas database. 13 (38.2%) in alcohol-use disorder, one (2.9%) in anorexia nervosa, five (14.7%) in anxiety disorder, nine (26.5%) in attention-deficit/hyperactivity disorder, six (17.6%) in autism spectrum disorder, 13 (38.2%) in bipolar disorder, 16 (47.1%) in major depressive disorder, two (5.9%) in post-traumatic stress disorder, and 18 (52.9%) in schizophrenia. * indicates common genomic variant associations, and & indicates rare protein-coding variant associations; symbols are shown only where both variant types were identified for a given disorder.

### Genes harbouring dnSNVs in French-Canadian childhood-onset OCD probands display complete overlap with genes associated with other psychiatric disorders

No OCD-specific gene was identified as all 34 of our candidate genes overlapped with genes previously associated with one or more related psychiatric disorders. 10 of our 34 (29.4%) candidate genes were previously implicated in four related psychiatric disorders (autism spectrum disorder, intellectual disability, schizophrenia, and Tourette’s syndrome) by other DNV analyses (Table 2) [32]. Additionally, all 34 genes (100%) overlapped with loci found in GWAS of nine related psychiatric disorders (alcohol-use disorder; anorexia nervosa; anxiety disorder; attention-deficit/hyperactivity disorder; autism spectrum disorder; bipolar disorder; major depressive disorder; post-traumatic stress disorder; and schizophrenia) (Table 2) [33].

### Functional enrichment meta-analyses of genes harbouring DNVs in childhood-onset OCD probands

**Fig. 2:**
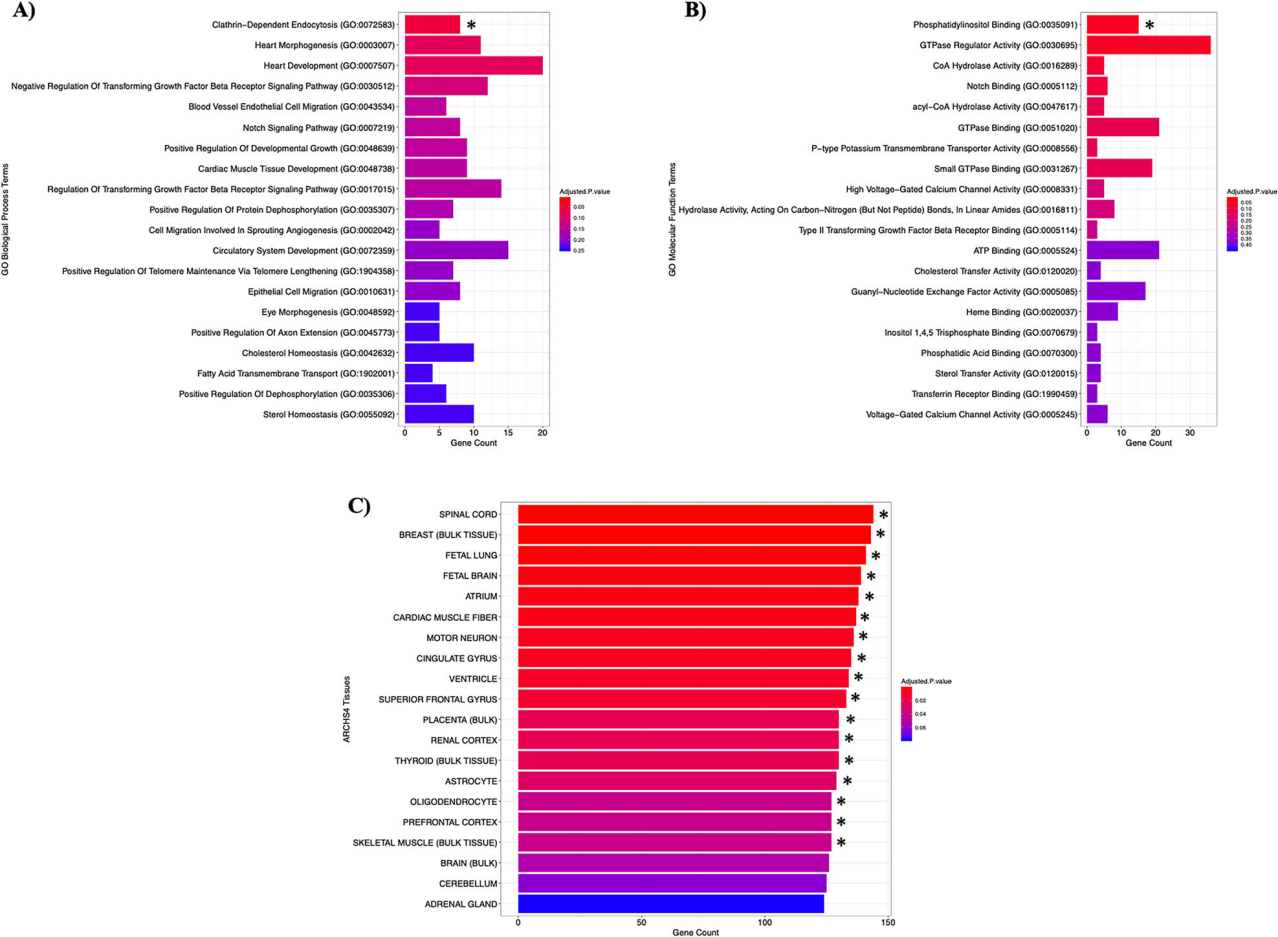
Functional enrichment meta-analyses of genes harbouring DNVs in childhood-onset OCD probands using EnrichR. The input candidate gene list included the 34 genes harbouring dnSNVs in the French-Canadian probands from the present study as well as 871 genes harbouring DNVs in two previous childhood-onset OCD publications (228 genes from Cappi et al., 2020 [11]; 643 genes from Halvorsen et al., 2021 [12]) (*n* total = 905 genes). *P*-values were adjusted for multiple comparisons using the BH-FDR method. * denotes a significant enrichment after BH-FDR correction (*p-adj* < 0.05). Significant term enrichments were confirmed using g:Profiler g:GOSt. **A** Enrichment of GO biological process terms. **B** Enrichment of GO molecular function terms. **C** Enrichment of ARCHS^4^ tissue terms.

Among genes harbouring DNVs across the three childhood-onset OCD DNV analyses (*n* = 905), we observed that the clathrin-dependent endocytosis GO biological process term (GO:0072583) was significantly overrepresented with eight genes related to that pathway harbouring DNVs across the three studies (*p-adj* = 0.0498) (Fig. 2A) [37]. We also observed that the phosphatidylinositol binding GO molecular function term (GO:0035091) was significantly overrepresented with 15 genes related to that pathway harbouring DNVs across the three studies (*p-adj* = 0.0431) (Fig. 2B) [37]. The combined list of candidate childhood-onset OCD genes also displayed a significant enrichment of genes expressed in various brain tissues and cells [38]. There was a significant overrepresentation of genes expressed in the spinal cord (*p-adj* = 0.000870); fetal brain (*p-adj* = 0.00267); motor neurons (*p-adj* = 0.00511); cingulate gyrus (*p-adj* = 0.00649); superior frontal gyrus (*p-adj* = 0.0106); astrocytes (*p-adj* = 0.0276); oligodendrocytes (*p-adj* = 0.0411); and prefrontal cortex (*p-adj* = 0.0411) (Fig. 2C) [38].

### Relationship between dnSNVs, parental age, proband age at OCD diagnosis, and symptom severity in childhood-onset OCD

We modelled the effect of parental ages at proband conception on the number of dnSNVs in French-Canadian childhood-onset OCD probands using two linear regression models. No association was found between the number of dnSNVs in probands and paternal age at proband conception (*p-adj* = 0.693, f^2^ = 0.592) or maternal age at proband conception (*p-adj* = 0.649, f^2^ = 0.618) (Table 3A). Next, we modelled the effect of the number of dnSNVs in our probands on proband age at OCD diagnosis using a linear regression model. No association was found between the number of dnSNVs in probands and proband age at OCD diagnosis (*p-adj* = 0.811, f^2^ = 0.507) (Table 3B). We then modelled the effect of the number of dnSNVs in French-Canadian childhood-onset OCD probands on whether OCD symptoms were rated as severe or mild to moderate using a logistic regression model. No association was found between the number of dnSNVs in probands and OCD symptom severity (*p-adj* = 0.379, f^2^ = 0.900) (Table 3C). Additionally, there was no association identified between OCD symptom severity and whether probands had first-degree relatives with a psychiatric diagnosis (*p-adj* = 0.379, f^2^ = 0.900); history of traumatic brain injury (*p-adj* = 0.643, f^2^ = 0.900); or presence of neuropsychiatric comorbidities (*p-adj* = 0.379, f^2^ = 0.900) (Table 3C). Post hoc power analyses (α = 0.05, β = 0.8) for each model (N = 32, 31, 36, and 30, respectively) indicated large detectable effect sizes (f² > 0.35).

**Table 3:** Relationship between the number of dnSNVs in French-Canadian childhood-onset OCD probands and clinical outcomes.

A) Modeling the effects of parental ages at conception on the number of dnSNVs observed in probands						
	Paternal age at conception			Maternal age at conception		
Predictor	Est. [95%CI]	SE	*p-adj*	Est. [95%CI]	SE	*p-adj*
Parental age	−0.0416 [−0.112, 0.0291]	0.0361	0.693	−0.0662 [−0.174, 0.0421]	0.0552	0.649
Proband sex	0.0477 [−0.682, 0.778]	0.372	0.899	0.142 [−0.597, 0.882]	0.377	0.914
PC1	0.515 [−1.53, 2.56]	1.04	0.898	0.869 [−1.26, 3.00]	1.09	0.750
PC2	0.138 [−1.84, 2.12]	1.01	0.899	0.225 [−1.78, 2.23]	1.02	0.914
PC3	−0.439 [−2.46, 1.58]	1.03	0.898	−0.117 [−2.22, 1.98]	1.07	0.914
PC4	0.596 [−1.38, 2.57]	1.01	0.898	0.750 [−1.24, 2.74]	1.02	0.750
PC5	−1.45 [−3.43, 0.531]	1.01	0.657	−1.49 [−3.47, 0.489]	1.01	0.614

B) Modeling the effect of the number of dnSNVs observed in probands on proband age at OCD diagnosis			
Predictor	Est. [95%CI]	SE	*p-adj*
dnSNV number	−0.323 [−1.22, 0.578]	0.460	0.811
Proband sex	1.40 [−0.284, 3.09]	0.861	0.375
PC1	−1.62 [−6.56, 3.33]	2.52	0.811
PC2	−0.666 [−5.63, 4.30]	2.53	0.908
PC3	−1.34 [−6.41, 3.73]	2.59	0.811
PC4	0.120 [−4.85, 5.09]	2.54	0.963
PC5	−3.80 [−8.72, 1.11]	2.51	0.375

C) Modeling the effect of the number of dnSNVs observed in probands on OCD symptom severity			
Predictor	Est. [95%CI]	SE	*p-adj*
dnSNV number	−2.69 [−6.89, −0.564]	1.52	0.379
First-degree relatives	4.09 [0.197, 10.2]	2.43	0.379
Brain trauma	1.15 [−3.47, 6.97]	2.49	0.643
Comorbidity	3.21 [−0.857, 9.08]	2.45	0.379
Proband sex	−2.08 [−7.80, 1.26]	2.15	0.572
Proband age at diagnosis	0.673 [−0.0683, 2.02]	0.492	0.379
PC1	−14.3 [−-40.4, −1.05]	10.1	0.379
PC2	−1.84 [−11.0, 5.66]	3.90	0.643
PC3	4.42 [−7.36, 20.3]	6.74	0.615
PC4	2.74 [−4.30, 11.0]	3.53	0.615
PC5	−4.37 [−19.0, 7.42]	6.13	0.615

No significant association after BH-FDR correction (*p-adj* < 0.05) was detected in any of the models. **A)** Two linear regressions modeling the effect of parental age at proband conception on the number of dnSNVs observed in French-Canadian childhood-onset OCD probands. 4 probands without paternal birthdate information were excluded from the first linear regression. 5 probands without maternal birthdate information were excluded from the second linear regression. **B)** A linear regression modeling the effect of the number of dnSNVs in French-Canadian childhood-onset OCD probands on age at OCD diagnosis. All 36 probands were included in the analysis. **C)** A logistic regression modeling the effect of the number of dnSNVs in French-Canadian childhood-onset probands on OCD symptom severity measured using CY-BOCS scores. 6 probands without CY-BOCS data were excluded from the analysis.

## Discussion

This is the first DNV analysis of childhood-onset OCD in the French-Canadian population of Québec, Canada. This study provides the first estimate of the de novo mutation rate as well as the ratio of non-synonymous to synonymous dnSNVs in French Canadians with childhood-onset OCD. The estimated de novo mutation rate of 1.40 × 10^−8^ per nucleotide per generation for our cohort falls within the expected dnSNV rate range of 1.0–1.8 × 10^−8^ per nucleotide per generation [7]. This was not surprising as cases of childhood-onset OCD are not expected to have a higher rate of dnSNVs compared to unaffected controls per se. Rather, it is expected that the dnSNVs observed in childhood-onset OCD probands will be more deleterious than those observed in unaffected individuals. The observed ratio of non-synonymous to synonymous dnSNVs of 1.43:1 in our probands is slightly lower than the expected ratio of 2.23:1 reported by previous studies [40]. We propose two explanations for this observation. Firstly, it is possible that this lower-than-expected ratio occurred by chance due to our small sample size. Alternatively, it could point to synonymous dnSNVs as an important source of variation in childhood-onset OCD risk. Future studies will be needed to elucidate the role of synonymous dnSNVs in childhood-onset OCD.

Using our framework, we identified 34 genes that harboured potential childhood-onset OCD-associated dnSNVs specific to the French-Canadian population. The individual expression of all 34 candidate genes in various brain tissues of GTEx Project samples provides further confidence for their potential roles in childhood-onset OCD risk [34]. The four genes (i.e., *INTS1*, *TIFAB*, *SPATA6*, and *ZNF669*) previously associated to OCD [12, 31] are the most promising candidate genes of childhood-onset OCD identified in this study due to those repeated observations in unrelated probands. The other 30 genes potentially represent novel childhood-onset OCD candidate genes to be screened in OCD cohorts of various origins. The 13 genes harbouring dnSNVs with CADD scores > 20 also represent good candidates for follow-up investigation in childhood-onset OCD due to the predicted severity of these variants [28].

We did not identify any dnSNVs in genes that were uniquely associated with childhood-onset OCD. This finding supports an overlapping genetic architecture between childhood-onset OCD and 11 related psychiatric disorders (alcohol-use disorder; anorexia nervosa; anxiety disorder; attention deficit/hyperactivity disorder; autism spectrum disorder; bipolar disorder; intellectual disability; major depressive disorder; post-traumatic stress disorder; schizophrenia; and Tourette’s syndrome) [41]. This implies a potential pleiotropic effect and supports the hypothesis of a shared underlying genetic susceptibility across psychiatric disorders. This finding is in line with previous studies which were also unable to identify any OCD-specific genes [41].

We observed a nominally significant genome-wide excess of splice site dnSNVs within our cohort, suggesting alternative splicing may be an important mechanism in the pathogenesis of childhood-onset OCD. Interestingly, the two high-confidence OCD risk genes identified by previous DNV analyses, *CHD8* and *SCUBE1*, each harboured a splice site dnSNV predicted to decrease splicing efficiency in unrelated childhood-onset OCD probands [11, 12]. These results support our ongoing hypothesis of alternative splicing as a potential risk factor in childhood-onset OCD. While future analyses will be needed to confirm and clarify the role of splice site dnSNVs in childhood-onset OCD risk, this study provides the first evidence of an enrichment of rare splice site variants in childhood-onset OCD probands.

Among genes harbouring DNVs across the three DNV analyses of childhood-onset OCD [11, 12], genes involved in clathrin-dependent endocytosis (GO:0072583) and phosphatidylinositol binding (GO:0035091) were enriched [35–37]. Clathrin-dependent endocytosis is a fundamental cellular process whereby cargo molecules undergo internalization from the cell surface and are subsequently transported into vesicles within the cell [42]. It is a particularly essential pathway for proper neural functions [42]. Aberrant clathrin-dependent processes have been associated with the pathogeneses of several psychiatric disorders, including bipolar disorder and schizophrenia, as well as neurodegenerative disorders, such as Parkinson’s disease [42–44]. Phosphatidylinositol consists of a family of phospholipids, known as phosphoinositides, that play key roles in cell physiology and signaling [45]. Dysfunction of the phosphatidylinositol pathway has been implicated in and suggested as a therapeutic target for OCD [46, 47]. Of note, phosphatidylinositol 4,5-bisphosphate is the main lipid binding partner of proteins involved in clathrin-dependent endocytosis [48], suggesting that aberrant phosphatidylinositol binding may lead to the dysfunction of clathrin-dependent endocytosis processes in OCD pathogeneses. However, more research will be needed to validate the involvement of these two biological pathways in childhood-onset OCD. Nonetheless, this study provides the first evidence of their dysregulation as potential genetic risk factors for the disorder.

Our functional enrichment meta-analyses also revealed an overrepresentation of genes expressed in various brain tissues and cells [11, 12, 35, 36, 38]. Most interestingly, we found an enrichment of genes involved in the fetal brain [38], suggesting that genes harbouring DNVs in childhood-onset OCD probands may also play a role in neurodevelopment. This is supported by the observed enrichment of genes expressed in astrocytes and oligodendrocytes [38], two key cells in the mammalian brain with crucial roles in neurodevelopmental processes [49, 50]. Also of note, we observed an overrepresentation of genes expressed in the cingulate gyrus and the prefrontal cortex [38], two key components of the cortico-striatal-thalamo-cortical (CSTC) system [51]. The CSTC neural loop is involved in many cognitive and emotional processes, including reward-based learning, decision making, and goal-directed behaviour [51]. It is also involved in important motor functions, including procedural and habit learning, the selection and execution of appropriate actions, action inhibition, and impulsivity control [51]. The CSTC has been consistently implicated in obsessive-compulsive tendencies and its dysfunction is widely hypothesized to underlie OCD [1, 51]. Altogether, our findings support two ongoing hypotheses: that childhood-onset OCD is a potential neurodevelopmental subtype of the disorder [52], and that the CTSC system is a key neural loop involved in the pathogenesis of OCD [1, 51].

There exist several potential limitations to the present study. Our sample size is small for a DNV analysis, limiting our statistical power to detect exome-wide significant genes as well as statistically significant associations within our regression models. Most notably, the linear regression models investigating the effect of advanced parental age at proband conception on the number of dnSNVs detected in our probands were likely underpowered due to our small sample size given it is widely recognized that advanced paternal age is associated with an increased de novo mutation rate [53]. Our sample size also calls for follow-up analyses with larger cohorts to confirm and clarify our findings. Moreover, many of our probands have neuropsychiatric comorbidities (including tic, anxiety, eating, depressive, social, and language disorders). Consequently, some of the genes identified in this study may represent those contributing to multiple comorbid neuropsychiatric disorders or to the general risk for psychopathologies rather than childhood-onset OCD specifically. Furthermore, without access to age- and ancestry-matched control trios, an enrichment analysis of dnSNVs in cases compared to controls could not be performed to support the role of dnSNVs in childhood-onset OCD for French Canadians. The use of self-reported ancestry may also represent a limitation as self-reports can be inaccurate for determining genetic ancestry given they are often influenced by non-genetic information [54]. While QC steps were implemented to ensure a genetically homogenous cohort indicative of French-Canadian genetic ancestry, it remains possible that some trios included in the analyses are of broader European ancestry. Additionally, by virtue of using WES, we were limited to the detection of dnSNVs located in the sequenced exome of our probands. Therefore, we were unable to detect dnSNVs located in non-coding regions that may be contributing to childhood-onset OCD risk. Finally, as in any DNV study, the possibility that there may be undetected dnSNVs in our available proband data relevant to our phenotype of interest cannot be excluded.

Nonetheless, this study presents a framework for conducting DNV analyses of complex psychiatric disorders in genetically isolated populations wherein sample sizes are often limited. Using this framework, we provided the first list of candidate childhood-onset OCD genes that are specific to the French-Canadian population. We also identified biological mechanisms that may be underlying childhood-onset OCD risk and provided further support for a shared underlying genetic susceptibility to psychiatric disorders. While larger sample sizes with deep phenotypic information will be needed to elucidate the role of dnSNVs in childhood-onset OCD symptom severity, we have provided a simple statistical model for investigating this relationship and emphasize the value of robust clinical data collection for these models. Altogether, this study sets the foundation for potential future studies with larger sample sizes that could afford greater resolution for detection via increased power of discovery. Future analyses should also focus on adult-onset OCD cases to better understand the role of DNVs in this patient subgroup.

## Supplementary information

Supplementary Figures

Supplementary Tables

## Data Availability

The data utilized in this study are available upon reasonable request to the corresponding author.

## Code availability

All codes used to generate results are available from the corresponding author upon request. QC was performed using GATK v3.8 and PLINK 2.0. Blacklist variants were filtered using the ENCODE Blacklist Regions GRCh37 v2 and bedtools v2.30.0. Kinship analysis and PCA were performed using SNPRelate v1.24.0. PCA also used tidyverse v1.3.2 and the 1000 Genomes Reference Panel GRCh37 Phase 3 v5a. DNV calls were annotated using the Ensembl VEP v107. The deleteriousness of the dnSNVs was scored using CADD GRCh37 v1.6. dnSNV MAFs were determined using gnomAD v.4.1.0. IGV v2.13.0 was used to visually inspect the dnSNVs. dnSNV enrichment by variant class was performed using denovolyzeR v0.2.0. Gene overlap was determined using the Gene4Denovo database v1.2 and the GWAS Atlas database v20191115. Gene expression levels in brain tissues were determined using GTEx v8. EnrichR v3.2 and g:Profiler g:GOSt v0.2.3 were used for the functional enrichment meta-analyses.
